# Cardiac and vascular structure and function parameters do not improve with alternate nightly home hemodialysis: An interventional cohort study

**DOI:** 10.1186/1471-2369-12-51

**Published:** 2011-10-03

**Authors:** Carolyn L van Eps, Leanne Jeffriess, Brian Haluska, Carmel M Hawley, Jeffrey Coombes, Aya Matsumoto, Janine K Jeffries, David W Johnson, Scott B Campbell, Nicole M Isbel, David W Mudge, Thomas Marwick

**Affiliations:** 1 Department of Cardiology, Princess Alexandra Hospital, Ipswich Rd, Brisbane, 4102, Australia; 2 Department of Nephrology Princess Alexandra Hospital, Ipswich Rd, Brisbane, 4102, Australia; 3 School of Medicine, University of Queensland, Ipswich Rd, Brisbane, 4102 Australia; 4 Department of Human Movements, University of Queensland, St Lucia, Brisbane, 4067, Australia

**Keywords:** Diastolic Function, Ejection Fraction, Left Ventricular Mass Index, Left Ventricular Hypertrophy, Nocturnal Hemodialysis, Carotid Intima-Media Thickness, Oxidative Stress, Arterial Compliance

## Abstract

**Background:**

Nightly extended hours hemodialysis may improve left ventricular hypertrophy and function and endothelial function but presents problems of sustainability and increased cost. The effect of alternate nightly home hemodialysis (NHD) on cardiovascular structure and function is not known.

**Methods:**

Sixty-three patients on standard hemodialysis (SHD: 3.5-6 hours/session, 3-5 sessions weekly) converted to NHD (6-10 hours/session overnight for 3-5 sessions weekly). 2Dimensional transthoracic echocardiography and ultrasound measures of brachial artery reactivity (BAR), carotid intima-media thickness (CIMT), total arterial compliance (TAC) and augmentation index (AIX) were performed post dialysis at baseline and 18-24 months following conversion to NHD. In 37 patients, indices of oxidative stress: plasma malonyldialdehyde (MDA) and anti-oxidant enzymes: catalase (CAT), glutathione peroxidase (GPX) and superoxide dismutase (SOD) activity and total antioxidant status (TAS) were measured at baseline, 3 and 6 months.

**Results:**

Left ventricular mass index (LVMI) remained stable. Despite significant derangement at baseline, there were no changes in diastolic function measures, CIMT, BAR and TAC. AIX increased. Conversion to NHD improved bone mineral metabolism parameters and blood pressure control. Interdialytic weight gains increased. No definite improvements in measures of oxidative stress were demonstrated.

**Conclusions:**

Despite improvement in uremic toxin levels and some cardiovascular risk factors, conversion to an alternate nightly NHD regimen did not improve cardiovascular structure and function. Continuing suboptimal control of uremic toxins and interdialytic weight gains may be a possible explanation. This study adds to the increasing uncertainty about the nature of improvement in cardiovascular parameters with conversion to intensive hemodialysis regimens. Future randomized controlled trials will be important to determine whether increases in dialysis session duration, frequency or both are most beneficial for improving cardiovascular disease whilst minimizing costs and the impact of dialysis on quality of life.

## Background

Cardiovascular disease is a leading cause of morbidity and mortality, accounting for approximately 30-40% of deaths in end stage kidney disease (ESKD) patients [1]. Left ventricular hypertrophy (LVH), dilatation and systolic and diastolic dysfunction are common and independently associated with mortality [2,3]. These changes are postulated to result from chronic volume overload (due to salt and water retention, chronic anemia and arteriovenous fistulae), pressure overload (due to hypertension, atherosclerosis, vascular and cardiac valvular calcification), metabolic (acidosis, malnutrition, inflammation and oxidative stress) and neuroendocrine factors (renin-angiotensin-aldosterone and sympathetic activation) [4,5].

Vascular disease occurs in two main forms: 1) arteriosclerosis with diffuse arterial wall dilatation, thickening, fibrosis and calcification resulting in stiffening and 2) atherosclerosis with abnormal endothelial function and patchy intimal plaques, causing abnormal regulation of vascular tone, fibrinolysis and smooth muscle proliferation with narrowing or obstruction of the arterial lumen. Increasing arterial stiffness raises pulse wave amplitude and velocity, causing reflected pressure waves from the periphery to be stronger and to arrive in the ascending aorta in systole rather than diastole, thus increasing systolic blood pressure and decreasing diastolic blood pressure. The resulting increased pressure load on the left ventricle (LV) during systole promotes LV hypertrophy and the reduced pressure in diastole reduces coronary artery perfusion, promoting myocardial ischemia [6,7]

Traditional risk factors for CV disease are more prevalent in ESKD patients compared to the general population. When adjusted for age, gender and race, ESKD patients have a higher prevalence of diabetes, hypertension, physical inactivity, hypertriglyceridemia and reduced high density lipoprotein [8]. However, traditional risk factors explain only approximately half the all cause mortality and variation in CV mortality in ESKD [9]. Other novel risk factors including inflammation, malnutrition, anemia, vascular calcification secondary to deranged bone mineral metabolism (BMM), oxidative stress and hyperhomocysteinemia have been associated with adverse CV outcomes in ESKD [10,11]. The exact role of these novel risk factors as surrogate markers of CV disease and mortality in ESKD remains controversial.

Daily nocturnal hemodialysis (6-7 nights weekly, 8-10 hours/session) has been associated in observational studies [12-18] and 1 randomized controlled trial [19], with significant, sustained improvement in surrogate markers of mortality including BMM indices, blood pressure, LV ejection fraction and mass, homocysteine, serum albumin and hemoglobin. One group has shown improvement in BAR and arterial compliance [20-22]

Alternate nightly hemodialysis(NHD) may reduce the burden and financial cost of dialysis compared with daily regimens, however, its effects on cardiovascular parameters are undefined. We sought to compare cardiovascular responses and cardiovascular risk factor profiles in standard duration hemodialysis (SHD) and alternate nightly NHD regimens, and to identify the correlates of these changes.

## Methods

### Study Population and Design

The study followed a prospective cohort design performed at a single centre. Between 2003 and 2006, consenting adults (≥ 18 years) with ESKD, established on home hemodialysis for ≥ 3 months were converted from a 3.5-6 hours/session, 3-5 sessions weekly home hemodialysis regimen to home NHD (6-10 hours overnight/session for 3-5 sessions weekly). Within the range of 3-5 sessions weekly and 6-10 hours/session, patients tailored their dialysis regimen to suit their lifestyle and sleeping habits. Interdialytic intervals of greater than 2 days were discouraged. Changes to the dialysis prescription are outlined in Tables 1 and 2

**Table 1:** Demographic and Baseline Clinical Data

Clinical Variable	NHD (n = 63)
**Age (Years)**	51.5 ± 13
**% Male**	79
**Body Mass Index(kg/m^2^)**	28.9 ± 7.6
**Dialysis Duration (%) < 2 years**	56
**2-5 years**	25
**> 5 years**	19
**Previous Renal Transplant(%)**	22
**Smoking Status (%) Never**	46
**Former**	48
**Current**	6
**Clinically Apparent Cardiovascular Disease(%)**	25
**Diabetes (%)**	33

NHD = Nocturnal hemodialysis

**Table 2:** Dialysis Prescription 1

Variable	Baseline		Nocturnal Haemodialysis	
**Frequency (sessions/week)**	3:	9.5%	3:	4.8%
	3.5:	47.6%	3.5:	69.4%
	4:	39.7%	4:	24.2%
	5:	3.2%	5:	1.6%
**Median (Interquartile Range)**	3.5 (3-4)		(3.5 (3.5-4)	
**Duration (Hours/Session)**	< 4:	6.3%	6-7.5:	9.7%
	4:	14.3%	8:	9.7%
	4.5:	12.7%	8.5:	14.5%
	5:	41.3%	9:	51.6%
	5-6:	25.3%	9-11:	14.5%
**Median (Interquartile Range)**	5 (3.5-5)		9 (7-10)	
**Membrane Type** **Surface Area (m^2^)**	Polysulfone		Polysulfone	
	1.4:	1.6%	1.4:	16.1%
	1.7:	52.4%	1.7:	56.5%
	2.1:	46.0%	2.1	27.4%
**Median (Interquartile Range)**	1.7	(1.7-2.1)	1.7	(1.4-2.1)
**Flux**	High:	95.2%	High:	100%
**Blood Flow Rate (mL/min)**	200-230:	3.2%	200-230:	43.5%
	240-275:	27.0%	240-275:	53.2%
	280-325:	49.2%	280-325:	3.3%
	330-360:	20.6%	330-360:	
**Median (Interquartile Range)**	275 (250-350)		250 (200-275)	
**Dialysate Flow Rate (mL/min)**			300:	20.6%
	500:	100%	500:	79.4%
**Cannulation Technique**	Self Cannulating: 76.2%		Self Cannulating: 76.2%	
	Dual Needle (14/15 guage)		Dual Needle (14/15 guage)	
	AVF: 90.5% Buttonhole		AVF: 93.7% Buttonhole	
	AVG: 100% Rope Ladder		AVG: 100% Rope Ladder	

NHD = Nocturnal hemodialysis

A diet low in saturated fat, salt and sugar with a daily protein intake of 1.2-1.4 g/kg dry weight/day and fluid restriction to maintain interdialytic weight gains < 3 kg was recommended. Dietary phosphate and potassium restrictions were relaxed upon commencement of NHD. Phosphate binders were ceased at the time of conversion to NHD and reintroduced only as required to maintain predialysis serum phosphate < 1.6 mmol/L. At baseline, calcitriol^® ^(1,25 OH_2 _Vit D) was only continued in patients in whom it was required to maintain serum calcium in the normal range post previous parathyroidectomy or in those with severe hyperparathyroidism (PTH > 8 times the upper limit of normal). Vitamin D supplements were reintroduced as necessary to maintain predialysis serum calcium levels in the normal range and parathyroid hormone levels 2-6 times the upper limit of the normal range where possible. All patients were prescribed folic acid 5 mg daily, Multi B Forte ^® ^1 tablet daily (Thiamine 7.27 mg, Riboflavin 6 mg, Nicotinamide 45 mg, pyridoxine 0.7 mg and ascorbic acid 45 mg) and pyridoxine 25 mg daily as oral supplements. The protocol for conversion to NHD was approved by the Princess Alexandra Hospital Human Research Ethics Committee.

### Outcome measures

Cardiovascular structure and function studies were scheduled to be performed at baseline, 6, 12, 18 and 24 months following conversion to NHD. Cardiac and vascular studies at each time point were performed at the same visit. Images were collected post-dialysis and interpreted by experienced staff, blinded to patients' clinical information.

#### Echocardiographic Assessment

Left ventricular volumes, mass, systolic function, diastolic function and filling pressures were assessed using 2 Dimensional echocardiography. Left ventricular (LV) end diastolic (EDV) and end systolic volumes (ESV) were calculated using the Modified Simpson's Rule. LV mass was calculated using the Devereux method [23]. Left ventricular mass index (LVMI) was calculated by indexing LVMass to Height^2.7^, which is a better predictor of mortality in dialysis patients compared to LVmass indexed to body surface area (BSA) [24]. LVMI corrected to body surface area (Dubois and Dubois Method [25]) is also reported.

Diastolic function was assessed using peak early mitral inflow filling velocity (E), peak mitral filling velocity at atrial contraction (A), E/A, deceleration time of the mitral E wave (DT) and tissue doppler velocity of the mitral annulus (E^l^)[26]. LV filling pressures were assessed using the E/E^l ^ratio and left atrial volume (LAVol), calculated using the Modified Simpson's Rule. LAvol index (LAVolI) was calculated by indexing LaVol to BSA (Dubois and Dubois Method). The categorical definition of diastolic dysfunction was that outlined by Omen and Nishimura [26].

### Vascular Structure and Function Studies

Brachial Artery Reactivity (BAR), a measure of endothelial function, was calculated as percentage change in brachial artery diameter, measured using high resolution B mode ultrasonography, with the induction of reactive hyperemia induced by arterial occlusion for 4.5 minutes, using a sphygmomanometer cuff [27,28]. BAR in healthy populations has been reported to range from 0.2 to 19.2% [29]. Endothelial dysfunction measured by these methods has been shown to correlate with the extent and severity of coronary artery disease determined by angiography [30].

Carotid intima-media thickness (CIMT), a measure of arterial wall thickness, was determined by averaging 3 measurements taken on each carotid artery (anteriorly, laterally and posteriorly) measuring the distance between the leading edge of the lumen-intima interface and the leading edge of the collagenous upper layer of the adventitia using high resolution B mode ultrasonography. Measures were taken in areas free of obvious atherosclerotic plaque around the level of the carotid bifurcation. CIMT is a marker for the presence and severity of arteriosclerosis and has been associated with risk factors for cardiovascular disease, all cause and cardiovascular mortality [31-33] A systematic review has shown an increased risk of myocardial infarction with CIMT > 0.822 mm and an increased risk of stroke with CIMT > 0.75 mm [34]. Our group has shown increased cardiovascular and all-cause mortality is associated with CIMT > 0.62 mm [35].

Total arterial compliance (TAC), a measure of arterial stiffness, was calculated using the pulse-pressure method [36,37]. Applanation tonometry (Millar SPT-301 Mikro-Tip transducer, Millar Instruments, Houston, TX), was performed on the left radial artery. BP was measured in the right arm, using a sphygmomanometer, with the patient resting supine for 10 minutes. The radial tonometric waveform was calibrated by assuming equivalence of mean [(2*Diastolic BP + Systolic BP)/3] and diastolic brachial cuff pressure. The radial tonometric waveforms were obtained simultaneously with pulsed-wave Doppler, digitized (WaveBook 512, IOTech Inc., Cleveland, OH), and transferred to a computer, where they were synchronized using the R wave of the electrocardiogram. Using specialized acquisition software, ECG gated data using tonometry and Doppler were acquired. Echocardiographic images and pulsed Doppler were acquired using a standard ultrasound system with 3.5 MHz and 11 MHz harmonic imaging probes. Stroke volume was derived from the pulsed Doppler and LV outflow tract dimensions [38]. The tonometric waveforms and aortic outflow data were analysed using a custom written program. Up to 10 cardiac cycles were averaged and central pressure was derived by applying a generalized transfer function to the radial tonometric pressure data [39] Values for TAC were then derived using the iterative method described by Stergioupulos [40]. Our group has previously documented TAC in normal subjects of 1.32 ± 0.58 mL/mmHg([41]. Our group has also shown TAC < 0.94 mL/mmHg is associated with mortality and the composite endpoint of death and hospital admissions for cardiovascular causes [42].

Augmentation Index (AIX), which reflects cardiac stroke volume and the effects of vascular stiffness, was calculated using the formula: AIX = augmentation pressure/pulse pressure)*100, where augmentation pressure = systolic blood pressure -pressure at the first inflection point on the central pressure wave form which was generated using a validated generalized transfer function from recordings of the radial artery pulse using applanation tonometry (SphygmoCor 7.01; AtCor Medical) and pulse pressure = systolic-diastolic blood pressure [43,44]. Previously published studies have measured a central AIX in normal populations of 30-45% [45].

### Cardiovascular Risk Factors and Prescribed Medications

Interdialytic weight gains were calculated from pre and post dialysis total body weights measured by patients on bathroom scales. BP was recorded pre- and post-dialysis by patients using digital sphygmomanometers. Serum or plasma levels of lipids, hemoglobin, C reactive protein (CRP), albumin, parathyroid hormone (PTH), calcium, phosphate and homocysteine were measured using standard laboratory techniques on blood samples taken predialysis in the fasting state. Measures of oxidative stress including, plasma malonyldialdehyde (MDA) and red blood cell (RBC) anti-oxidant enzymes catalase (CAT), glutathione peroxidase (GPX) and superoxide dismutase (SOD) activity, and plasma GPX activity and total antioxidant status (TAS) were measured pre and post dialysis at baseline and 3 and 6 months after conversion to NHD, in the last 37 patients recruited to the study. MDA was measured using high performance liquid chromatography. Activities of CAT, GPX and SOD and TAS were measured using standard spectrophotometric methods. Prescribed medications were determined by review of the medical record and patient interview.

#### Statistical methods

Statistical analyses were performed using STATA SE version 10.0. Sample size calculations indicated that 21 patients were required to have 80% power of detecting a 30% decrease in LVMass, allowing for 33% drop-out rate. Fifteen patients were required to have 80% power to detect a 4% change in BAR; 29 patients to detect a 0.05 mm change in CIMT and 13 patients to detect a 0.4 unit change in TAC with conversion to NHD.

Variables are expressed as percentages for categorical data and, for continuous data, as mean ± standard deviation if normally distributed and median (interquartile range) if skewed. Longitudinal analysis was performed using paired t-test if normally distributed and Wilcoxon signed rank test if skewed. Univariable and multivariable linear regression analyses were performed to assess correlations between changes in cardiovascular parameters and risk factors. The covariates considered for inclusion in the model included age, gender, duration of renal replacement therapy, diabetes and changes in lipids, systolic and diastolic blood pressure, interdialytic weight gains, parathyroid hormone, phosphate and calcium, hemoglobin, ferritin and transferrin saturation, C reactive protein and albumin. Those with a p < 0.25 on univariable analysis were introduced into a multivariable model. Then a stepwise, backward elimination process was performed, removing variables which contributed least significantly to the model provided they did not change the incident rate ratio by more than approximately 10%.

For correlation analyses, results are reported as the point estimate, β and 95% confidence intervals (CI), the correlation coefficient (r) and p value. P value < 0.05 was considered significant *a priori*.

## Results

### Demographic Data and Dialysis Prescription

Eighty-seven percent of the eligible home hemodialysis population agreed to participate in the study. Demographic and dialysis prescription data are presented in Tables 1 and 3. Median kt/V urea was 1.3 (1.1-1.5) on conventional hemodialysis and 1.5 (1.3-1.9) on NHD.

**Table 3:** Dialysis Prescription 2

Variable	Baseline		Nocturnal Haemodialysis	
**Dialysate Calcium (mmol/L)**	0.9-1.0:	17.5%		
	1.25-1.3:	77.8%	1.25-1.3:	1.6%
	1.5-1.6:	4.7%	1.5-1.6:	90.3%
	1.75:		1.75:	8.1%
**Median (Interquartile Range)**	1.3 (1.0-1.3)		1.5 (1.5-1.75)	
**Dialysate Potassium (mmol/L)**	1-1.25:	19.0%	1-1.25:	32.3%
	1.5:	47.7%	1.5:	21.0%
	2.0-2.5:	27.0%	2.0-2.5:	43.5%
	3:	6.3%	3:	3.2%
**Median (Interquartile Range)**	1.5 (1.25-3)		1.5 (1-2.5)	
**Dialysate Bicarbonate (mmol/L)**			28-30:	16.1%
	32:	3.2%	32:	83.9%
	35:	96.8%		
**Dialysate Sodium (mmol/L)**	140:	100%	140:	100%
**Dialysate Magnesium (mmol/L)**	0.5:	100%	0.5:	100%
**Dialysate Glucose (mmol/L)**	5:	100%	5:	100%
**Dialysate Phosphate**	0:	100%	30.6% added phosphate in the form of fleet 0-40 mL	

NHD = Nocturnal hemodialysis

Four patients died (Followed on NHD for 2, 5, 12 and 17 months)), 10 received renal transplants (Followed on NHD for 7.5 [6-17] months) and 7 withdrew consent to participate in the study (Followed on NHD for: 9 [2-13] months) during the 24 months follow up. The other patients without paired data for analysis failed to attend for investigations without withdrawing from the study.

Follow-up cardiac and vascular studies were performed at 18 (12-24) months. The timing of follow up investigations was determined by patient attendance at echocardiograms that were scheduled to occur 6 monthly between baseline and 24 months following conversion to NHD. The majority of patients only attended for 2 echocardiograms. The study performed at the latest time point during the 24 month follow up was utilized for analysis. Paired data was available for echocardiographic parameters in 38 patients, CIMT and BAR in 42 patients and TAC and AIX in 28 patients.

### LV Structure and Function

**Figure 1:**
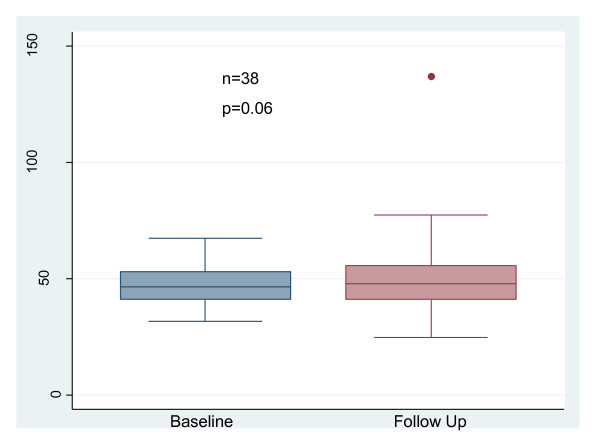
**Change in Left Ventricular Mass Index with Conversion to NHD**. NHD = Nocturnal hemodialysis.

Measures of LV structure and systolic function did not change between baseline and follow up. (Table 4 and Figure 1) At baseline, 64% of patients had LVMI within the normal range. Of the 40 patients with paired data, 93% maintained LVMI within 30% of baseline measurement and only 7% had a ≥ 30% increase in LVMI. In the subset of patients with abnormal LVMI at baseline, there was no change in LVMI over time (n = 14, 57 ± 6 vs 61 ± 10 g/m^2.7^).

**Table 4:** Change in Left Ventricular Structure and Systolic and Diastolic Function with Conversion from SHD to NHD

Variable	Baseline on SHD	Follow up on NHD	Comparison (Δ (95% CI), p value) (n = 38)
**EDV (mL)** **(Normal Mean: Men 111 mL, Women 80 mL)**	114 ± 28	112 ± 40	-4.37 (-14.01 to 5.27) p = 0.4
**ESV (mL)** **(Normal Mean: Men 34 mL, Women 29 mL)**	47 ± 14	50 ± 26	0.98 (-4.28 to 6.23) p = 0.7
**LVEF (%)** **(Normal > 50%)**	59 ± 6	57 ± 8	1.71 (-4.28 to 3.95) p = 0.1
**LVMI (g/m2.7) (Cut off for LVH: men 50, Women 47 g/m2.7)**	47 ± 10	49 ± 12	2.34 (-0.08 to 4.76) p = 0.06
**Early Transmitral Flow Velocity (m/s)** **(Normal 0.6-0.8 m/s)**	0.80 ± 0.25	0.79 ± 0.28	-0.005 (-0.08 to 0.07) p = 0.88
**E/A Ratio (Normal < 0.75)**	1.0 ± 0.49	1.06 ± 0.37	0.02 (-0.12 to 0.16) p = 0.77
**Deceleration Time (ms) (Normal > 240 ms)**	227 ± 57	213 ± 48	-14.10 (-28.79 to 0.61) p = 0.06
**Annular Tissue Doppler Velocity (cm/s) (Normal > 10 cm/s)**	5.88 ± 1.72	5.73 ± 1.85	-0.15 (-0.81 to -.52) p = 0.66
**E/Ei (Normal < 8)**	14 ± 4	15 ± 7	0.01 (-0.008 to 0.028) p = 0.28
**Left Atrial Volume Index (mL/m^2^) (Normal Median 21 mL/m^2^)**	40 ± 12	40 ± 12	-0.45 (-4.16 to 3.26) p = 0.81

SHD = Standard hours hemodialysis; NHD = Nocturnal hemodialysis; EDV = Left ventricular end diastolic volume; ESV = Left ventricular end systolic volume; LVEF = Left ventricular ejection fraction; LVMI = Left ventricular mass index; EA = Peak early mitral inflow filling velocity; A = peak mitral filling velocity at atrial contraction; E^l = Tissue Doppler ^velocity of the mitral annulus; CI = confidence interval

Measures of diastolic function and filling pressure remained stable (Table 4). Diastolic dysfunction was present in 74% of patients at baseline and 70% at follow up. The most common abnormality was raised LAVol.

### Vascular Structure and Function

**Figure 2:**
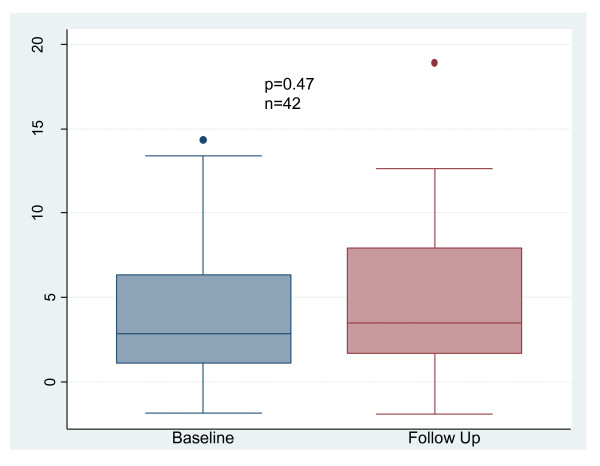
**Change in Brachial Artery Reactivity with Conversion to NHD**. NHD = Nocturnal hemodialysis.

**Figure 3:**
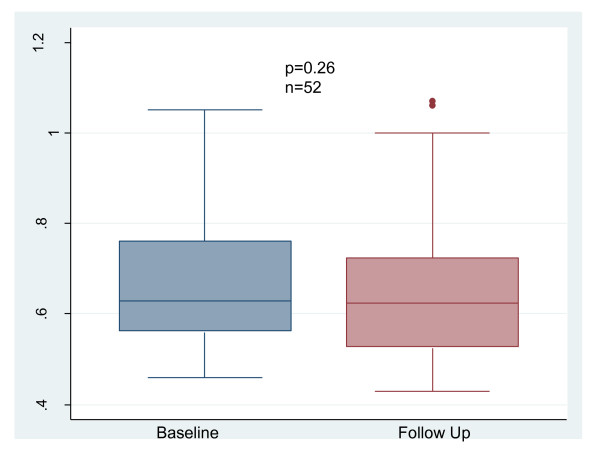
**Change in Carotid Intima Media Thickness with Conversion to NHD**. NHD = Nocturnal hemodialysis.

**Figure 4:**
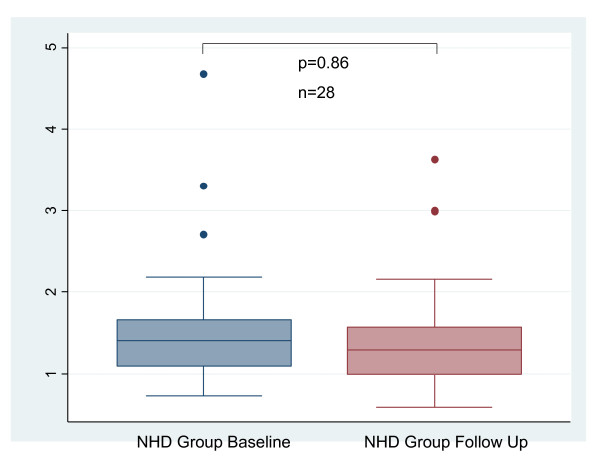
**Change in Total Arterial Compliance with Conversion to NHD**. NHD = Nocturnal hemodialysis.

**Figure 5:**
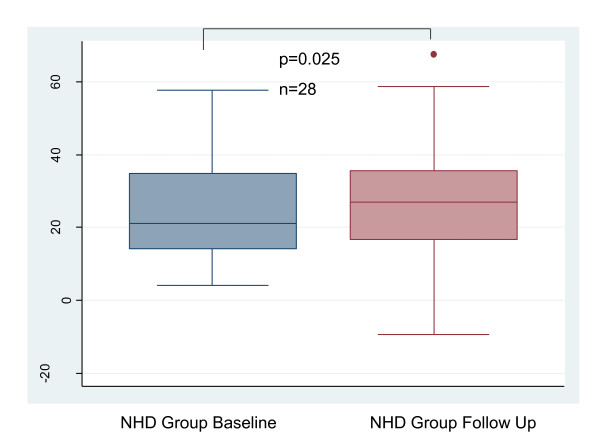
**Change in Augmentation Index with Conversion to NHD**. NHD = Nocturnal hemodialysis.

BAR did not change between baseline and follow-up. (Figure 2): 2.82 (1.12-6.3) vs 3.48 (1.68-7.95)%, p = 0.47. CIMT did not change between baseline and follow-up. (Figure 3): 0.66 ± 0.14 mm vs 0.65 ± 0.16 mm, p = 0.26. There was no significant change in TAC between baseline and follow-up: 1.4 (1.1-1.7) vs 1.3 (1.0-1.6)mL/mmHg, p = 0.86 (Figures 4). AIX increased from 21 (14-35)% to 27 (17-36)%, p = 0.025 (Figure 5). There was no significant change in pulse pressure between baseline and follow up: 66 (50-75) vs 60(50-73)mmHg, p = 0.46.

### Cardiovascular Risk Factors

Changes in cardiovascular risk factors are presented in Table 5. There was significant improvement in predialysis diastolic BP. There was a trend toward improvement in predialysis systolic BP. Interdialytic weight gains increased: 2.3 ± 0.8 vs 2.7 ± 1.0 kg, p = 0.006. Antihypertensive medication doses were stable or reduced in the majority (57% dose stable, 34% reduced, 9% increased). The percentage of patients being prescribed no antihypertensive medications increased from 47% to 66%, p = 0.03. Seven patients ceased an ACE inhibitor or angiotensin receptor blocker and 5 ceased a β blocker. One patient newly commenced an ACE inhibitor. HMG CoA reductase inhibitors were prescribed to 51% of patients at baseline. The dose was stable (65%) or reduced (3%) in the majority of patients and only 8% of patients newly commenced HMG CoA reductase inhibitor medications during the study. Hemoglobin and iron stores remained stable. Weekly darbepoietin alpha dose decreased (p = 0.007). Weekly intravenous iron dose remained stable.

**Table 5:** Changes in Vascular Risk Factor Profiles with conversion from SHD to NHD

Cardiac Risk Factor Parameter	NHD (n = 41)		
	**Baseline**	**Follow-up**	**p value**

**Total Cholesterol (mmol/L)**	4.4 ± 0.9	4.3 ± 0.8	0.8

**Triglycerides (mmol/L)**	1.6 (1.1-2.7)	1.1 (0.9-1.7)	0.08

**LDL Cholesterol (mmol/L)**	2.2 ± 0.8	2.1 ± 0.7	0.7

**HDL Cholesterol (mmol/L)**	1.5 ± 0.7	1.3 ± 0.4	0.08

**Lipoprotein a (mg/L)**	150 (90-260)	150 (80-278)	0.8

**Systolic Blood Pressure (mmHg)**	**148 ± 20**	**141 ± 19**	**0.06**

**Diastolic Blood Pressure (mmHg)**	**83 ± 15**	**78 ± 13**	**0.01**

**Haemoglobin (g/L)**	112 ± 12	114 ± 13	0.5

**Ferritin (mcg/L)**	209 (108-361)	253 (93-345)	0.5

**C Reactive Protein (mg/L)**	7(5-11)	6 (5-13)	0.6

**Albumin (g/L)**	42 ± 3	43 ± 4	0.2

**Calcium (mmol/L)**	**2.39 ± 0.30**	**2.48 ± 0.18**	**0.02**

**Phosphate (mmol/L)**	**1.93 ± 0.55**	**1.47 ± 0.35**	**< 0.0001**

**Calcium Phosphate Product (mmol^2^/L^2^)**	**4.61 ± 1.4**	**3.66 ± 0.86**	**< 0.0001**

**Parathyroid Hormone (pmol/L)**	**290 (140-471)**	**160 (62-290)**	**0.03**

**Homocysteine (mcmol/L)**	23 ± 6	22 ± 6	0.5

NHD = Nocturnal hemodialysis; LDL = Low density lipoprotein; HDL = High density lipoprotein

There was significant improvement in predialysis serum phosphate, calcium-phosphate product and parathyroid hormone levels (PTH). Serum predialysis corrected calcium levels rose. By 12 months following conversion to NHD, seventy-three percent of patients had predialysis serum phosphate levels ≤ 1.6 mmol/L. This was achieved despite a substantial decrease in requirement for phosphate binding medications (calcium carbonate, aluminium hydroxide and magnesium trisilicate). Only 6% of patients were not taking any phosphate binders at baseline and this increased to 79% on NHD. Calcium carbonate was prescribed to 90% of patients at baseline at an average dose of 2460 mg per day. On NHD, only 15% of patients were prescribed calcium carbonate at an average dose of 1670 mg/day. Addition of phosphate to the acid component of the dialysate on NHD was required in 29% of patients at an average dose of 20 ml of Fleet/10 L (Fleet contains sodium phosphate monobasic 19 g and sodium phosphate dibasic 7 g per 118 mL and sodium content = 4.4 g/118 mL). Parathyroid hormone levels fell significantly from an average baseline of 290 (140-471) to 160 (62-290) ng/L; p = 0.03. Fifty-eight percent of patients were prescribed calcitriol at baseline in an average dose of 1.4 mcg/week. After 12 months on NHD, 53% were prescribed calcitriol at an average dose of 1.8 mcg/week.

There was no significant change in predialysis plasma MDA. The increase in MDA observed during dialysis was attenuated after conversion to NHD. Predialysis RBC-CAT activity significantly increased between baseline and 6 months and change during dialysis remained stable. Predialysis RBC-GPX activity decreased and there was no change during dialysis. Plasma GPX activity increased marginally pre dialysis and had a greater increase during dialysis at 3 months. This was not sustained at 6 months. RBC-SOD and plasma TAS levels predialysis and change in these levels with dialysis remained stable (Table 6).

**Table 6:** Oxidative Stress Changes with Conversion to NHD

Variable	Pre dialysis	Post Dialysis	% Change with Dialysis
MDA (MicroM)			
Baseline	16.13+/-3.39	**18.1+/-3.74**	**3.3 (-3.8-27.6)**
3 Months	16.50+/-3.19	**16.24+/-2.51**	**-0.66(-8.70-12.41)**
6 Months	16.33+/-2.88	**16.4+/-2.94**	**-0.22 (-6.29-5.86)**
P value	0.43, 0.71	**0.015, 0.018**	**0.01, 0.005**

RBC CAT (U/mgHb)			
Baseline	**2.97+/-0.86**	**3.04+/-0.91**	2.7+/-12.5
3 Months	3.05+/-0.85	**3.21+/-0.82**	6.42+/-9.64
6 Months	**3.18+/-0.87**	**3.32+/-0.83**	5.47+/-10.80
P Value	0.18, **0.02**	**0.007, 0.002**	0.25, 0.40

RBC GPX (U/gHb)			
Baseline	**97.2 (40.1-113.4)**	**99.5 (41.3-112.0)**	1.53+/-7.16
3 Months	**83.4 (37.9-104.7)**	**81.1 (37.9-108.6)**	3.78+/-10.4
6 Months	**70.4 (37.2-95.0)**	**69.4 (36.0-92.9)**	-0.10+/-11.3
P Value	**0.0001, 0.0003**	**0.002, 0.0001**	0.37, 0.53

Plasma GPX (U/mL)			
Baseline	**0.030+/-0.008**	0.034+/-0.011	**12.8+/-11.1**
3 Months	**0.032+/-0.009**	0.035+/-0.011	**6.9+/-11.8**
6 Months	0.031+/-0.01	0.034+/-0.012	8.3+/-11.6
P Value	**0.04**, 0.35	0.79, 0.86	**0.02**, 0.13

RBC SOD (U/mgHb)			
Baseline	5.75+/-1.70	6.11+/-1.61	5.69+/-20.83
3 Months	6.32+/-2.30	6.02+/-1.58	-3.41+/-18.0
6 Months	5.87+/-1.63	5.72+/-1.76	4.00+/-30.74
P Value	0.29, 1.0	0.58, 0.33	0.08, 0.29

TAS			
Baseline	2.39+/-0.13	2.30+/-0.16	-4.06+/-4.47
3 Months	2.41+/-0.12	2.30+/-0.12	-4.69+/-2.89
6 Months	2.38+/-0.12	2.27+/-0.12	-4.55+/-3.21
P Value	0.54, 0.50	0.95, 0.33	0.64, 0.64

MDA = Plasma malonyldialdehyde; RBC = Red blood cell; CAT = Catalase; GPX = Glutathione peroxidase; SOD = Superoxide dismutase; TAS = Total antioxidant status

There were no strong associations between change in cardiovascular outcome measures and cardiovascular risk factors measured in this study. There were no significant associations on univariable or multivariable analysis with change in LVMI. Changes in diastolic parameters were associated on multivariable analysis with changes in markers of inflammation and nutrition, iron stores and PTH: early transmitral flow velocity and CRP: 0.008 (0.00001- 0.18), p = 0.05, R^2 ^= 0.48, E/A Ratio and CRP: 0.019 (0.003-0.04), p = 0.02, R^2 ^= 0.36, E/E^I ^and CRP: 0.003 (0.0005-0.005), p = 0.02, R^2 ^= 0.35 and E/E^I ^and Albumin: 0.006 (0.001-0.011), p = 0.02, R^2^0.35, early transmitral flow velocity and transferrin saturation: -0.008 (-0.13 to -0.003), p = 0.005, R^2 ^= 0.48, E/A ratio and transferrin saturation: -0.012 (-0.022 to -0.003) p = 0.01, R^2^0.36, deceleration time and ferritin: 0.08 (0.02-0.13), p = 0.01 R^2 ^= 0.49, early transmitral flow velocity and PTH: -0.0003 (-0.0006 to-4.01e^-6^), p = 0.05, R^2 ^= 0.48 and deceleration time and PTH: 0.05 (0.004-0.10), p = 0.02, R^2 ^= 0.49). LaVol Index was associated with interdialytic weight gain (9.47 (3.29-15.64), p = 0.004, R^2^0.45) on multivariable analysis.

Change in BAR was associated on multivariable analysis with age (-0.14 (-0.27 to -0.006), p = 0.04, R^2 ^= 0.32). Change in CIMT was associated on multivariable analysis with change in predialysis serum phosphate (0.34 (0.043-0.065), p = 0.03 and CRP (-0.002 (-0.004 to -0.003), p = 0.03, R^2 ^= 0.24). Change in AIX was associated on multivariable analysis with change in triglycerides (0.03 (0.001-0.06, p = 0.04 and CRP (0.01 (0.004-0.02), p = 0.003, R^2 ^= 0.52).

## Discussion

In this study, conversion from conventional home HD to alternate nightly home NHD had no significant effects on cardiovascular structure and function indices, after a mean follow-up of 18 months, despite favorable changes in recognized cardiovascular risk factors.

Previous studies examining the effects of using increased intensity dialysis or renal transplantation (RTx) to improve the uremic milieu, on cardiovascular structure and function have come to inconsistent conclusions. Some studies, including 1 randomized controlled trial (Culleton et al) have shown improvement in LV geometry with conversion to daily hemodialysis regimens (15, 17, 18, 19). The Frequent Hemodialysis Trial Group recently published important data from 2 randomized controlled trials. The first compared LVmass, measured by cardiac magnetic resonance imaging (MRI), in 125 patients managed with short daily hemodialysis with 120 patients managed on conventional thrice weekly hemodialysis [46]. Short daily hemodialysis resulted in a significant improvement in LV mass of 16.4+/-2.9 g compared to 2.6+/-3.2 g in the conventional hemodialysis group. In contrast, the second study compared LVmass, measured by cardiac MRI, in 87 patients randomized to either a daily nocturnal hemodialysis or a conventional thrice weekly hemodialysis regimen. This study showed no significant improvement in the primary outcome of death/LVmass composite [47]. Patel et al, found no improvement in LVMI 2.5 years after renal transplantation using cardiac MRI [48]

This study is the first to explore the effect of an extended hours hemodialysis regimen on cardiac diastolic function and no improvement was shown. Studies in renal transplantation, the gold standard for management of ESKD, have also consistently reported no improvement in diastolic dysfunction [49,50]. This may suggest that diastolic dysfunction associated with uremia may not be substantially reversible, even with correction of uremia and volume overload.

Chan et al reported significant improvement in BAR from -2.7 ± 1.8% to 8.0 ± 1.0% in 18 patients and TAC from 0.98 ± 0.13 to 1.43 ± 0.2 mL/mmHg in only 10 patients, 2 months following conversion to daily NHD. They have explored possible pathologic mechanisms driving this improvement and found increased baroreflex sensitivity for heart rate and proliferation and migration of endothelial and vascular smooth muscle cells with normalization of markers of vascular smooth muscle cell biology including caspase-3 and RunX-2 [20-22,51]. Several published studies show improvement, of approximately 1-4%, in endothelial function following renal transplantation using both non-invasive techniques, similar to those used in our study [52-55], and invasive techniques [56] at 2 weeks (n = 30), 1-3 months (n = 58, 27 and 42) and 12-24 months (n = 8). Studies examining the effect of RTx on CIMT reached inconsistent conclusions with some demonstrating improvement [55,57,58] (n = 42, 22 and 19, Follow-up = 3, 12 and 40 months) whilst others showed no improvement [59-61] (n = 36, 9 and 26, Follow-up: 12 and 6 months). Some [59,62] (n = 36 and 20, Follow-up = 12 and 3 months), although not all [63] (n = 36, Follow up = 3 months), studies have shown improvement in measures of arterial stiffness such as pulse wave velocity and AIX with RTx. AIX improved by approximately 10%.

One possible explanation for our findings is that patients in our study had lower baseline LVMI and less derangement of baseline BAR and TAC compared to patients in other quotidian hemodialysis studies where improvements were shown. Baseline LVMI indexed to BSA in our study was 106 ± 24 g/m^2^, appreciably lower than the baseline measurements reported by Ayus et al (154 ± 33 g/m^2^), Chan et al (180 ± 54 g/m^2 ^and 147 ± 42 g/m^2^) and Culleton et al (177.4 ± 51.1 g/m^2^) [14,17,19]. Some of these studies pre-selected patients with cardiovascular dysfunction for entry and were uncontrolled in design. Notably, our baseline dialysis schedule (3-5 sessions weekly, 3.5-6 hours/session) was more intensive than the regimens offered as standard therapy in many units and this may explain our excellent baseline LVMI. Although daily hemodialysis may improve LVMI, it may also increase the burden of dialysis for patients. With daily hemodialysis, the preparation and clean up time and the cost of consumables is doubled. Australia and New Zealand data shows a drift away from the use of daily hemodialysis regimens, with alternate daily NHD being the most popular and sustainable novel hemodialysis regimen [1]. Our data suggest that a 3.5-5 sessions weekly standard hours regimen may be sufficient to maintain normal LVMI in most patients. Regular echocardiographic surveillance may help identify a subset of patients with deteriorating LV parameters who may benefit from a trial of daily hemodialysis.

We note that in our study cardiac diastolic dysfunction and vascular structure and function parameters were significantly deranged at baseline in our population and did not improve with conversion to NHD. Conversion to alternate nightly NHD, which increased session duration but not frequency, did not improve LVH even in the subset of patients with high baseline LVMI. Therefore, an alternate explanation for the lack of improvement in cardiovascular parameters in our study may be inferior control of interdialytic weight gains (IDWG) and uremic toxins compared with the daily hemodialysis regimens or RTx. IDWG increased with conversion to alternate nightly NHD and may have negated any improvement associated with better control of other uremia toxins.

There is increasing literature supporting the hypothesis that all increased intensity hemodialysis regimens may not be associated with equal benefits to measures of cardiovascular structure and function. Both the FHN Trial group studies [47] and 1 published small, non-randomized study comparing alternate daily extended hours hemodialysis (n = 17) with short daily (n = 8) and standard conventional hemodialysis (n = 19)[64] have suggested that improvements in LVMass may be greater with short daily hemodialysis regimens compared to NHD. Possible reasons for this have not been adequately elucidated. It could be postulated that increasing session frequency maintains low IDWG. Prolongation of session duration may prolong the unfavorable hemodynamic changes that occur during hemodialysis, thus negatively effecting myocardial perfusion. In our study, only a minority of patients changed frequency of dialysis. Statistical adjustment for reduction in dialysis frequency when converting to NHD did not alter our results. More studies designed to examine the different effects of increasing session frequency as opposed to duration are needed to clarify these issues.

Previous studies in ESKD have found complex and inconsistent associations between proposed risk factors and cardiovascular disease [65,66]. Randomized controlled trials in ESKD have failed to show benefit from interventions correcting risk factors including anemia [67,68], dyslipidemia [69,70], hypertension, hyperhomocysteinemia and hyperphosphatemia [71]. In our study, conversion to NHD resulted in improved calcium, phosphate and PTH and a trend towards improved BP. Changes in markers of inflammation, iron stores and bone mineral metabolism were only weakly associated with changes in cardiovascular structure and function in this study. Our results highlight how poorly we understand the pathogenesis of vascular disease in ESKD and the need to explore novel risk factors which better explain, and provide therapeutic targets to reduce cardiovascular morbidity and mortality.

This study is the first to examine the effects of extended hours hemodialysis on oxidative stress. The procedure of hemodialysis has been postulated to increase oxidative stress through contact with bio-incompatible dialysis equipment and contaminated dialysis water, microhemolysis, alterations in lipid metabolism during heparin exposure and removal of antioxidants during dialysis [72]. Therefore it is possible that extended hours HD increases duration of exposure to these proinflammatory and oxidant stressors compared to conventional HD regimens. Contrary to this hypothesis, we documented attenuation of the increase in MDA during dialysis and no consistent increase in antioxidant enzyme activities. This suggests that, interestingly, there may be a reduction in lipid peroxidation during NHD compared to SHD. Inflammatory markers including CRP and WCC did not change with conversion to NHD. We documented an increase in predialysis RBC-CAT and a decrease in RBC-GPX with conversion to NHD. The increase in CAT, an antioxidant enzyme, may have caused the decrease in GPX, as both enzymes compete to convert peroxides to less oxidizing compounds. Predialysis MDA, a breakdown product of oxidized lipids and marker of oxidative stress [73] remained stable. It seems likely that interdialytic oxidative stress remained stable with conversion to NHD.

Vitamins C and E have been identified as important plasma antioxidants which may be lost in increased amounts during extended hours HD. We did not measure serum vitamin C or E levels, although vitamin C was routinely replaced in all patients. Vitamin C deficiency has been documented in extended hours dialysis patients where supplementation has not been routine [74]. This may have contributed to our failure to show more dramatic improvements in oxidative stress in our cohort. Further work is needed to confirm vitamin replacement requirements in NHD.

### Strengths and Weaknesses

This is the largest trial examining the effects of increasing dialysis duration on multiple measures of endothelial function and arterial wall thickness and stiffness and it is the only study examining cardiac diastolic function and oxidative stress markers. Echocardiograms and vascular ultrasound studies were all performed and interpreted in one experienced laboratory. Patients were all managed in one unit with uniform policies and procedures. We performed multivariable analyses to adjust for known confounders. We were powered to detect clinically significant changes in LVMI, BAR, TAC and CIMT.

However, our conclusions are limited by the non randomized study design. The baseline dialysis regimen was not a thrice weekly regimen as is standard in most facilities and may have been responsible for the more normal baseline LVMI in our study. It could be postulated that the observation periods used in our, and other studies have been too short to allow cardiovascular remodeling. 2Dimensional echocardiography is less precise and is more reliant on derived rather than directly measured values compared to cardiac magnetic resonance imaging for assessing LVMI [75]. Furthermore, the generally poor attendance at follow up study visits may have been a source of bias in this study. Approximately 1/3 of patients did not have follow-up studies completed. Despite these draw backs, we feel it has merit to assist with hypothesis generation, power calculations for randomized controlled trials and to guide clinical practice whilst definitive studies are conducted.

## Conclusion

Conversion to an alternate nightly home hemodialysis regimen, which only increased dialysis duration and not frequency, did not result in a significant improvement in cardiovascular structure and function. Continuing suboptimal control of uremic toxin levels, BP and fluid balance with alternate daily hemodialysis compared to daily hemodialysis or RTx may be a possible explanation. The relatively normal LVMI at baseline in our population also likely contributed to our failure to show improvement in this parameter. A wide range of traditional and novel cardiovascular risk factors did not strongly correlate with cardiovascular structure and function parameters.

This study adds to the increasing uncertainty in the published literature about the nature of improvement in cardiovascular parameters with conversion to intensive hemodialysis regimens. More work is needed to better understand the exact mechanisms driving cardiovascular dysfunction in ESKD and how we might prevent progression or reverse existing disease. Future randomized controlled trials will be important to determine whether increases in dialysis session duration, frequency or both are most beneficial for improving and slowing the progression of cardiovascular disease whilst minimizing costs and the impact of dialysis on quality of life.

## Competing interests

Authors on this paper have received travel and research grants and fees for lectures from Fresenius Medical Care, Baxter and Gambro. Carmel Hawley and David Johnson have been members of the advisory boards for Fresenius Medical care, Baxter and Gambro.

## Authors' contributions

CvE: Design, coordination, data collection cardiovascular risk profiles, recruitment, statistical analysis, drafting and revising the manuscript. LJ: Performed and analyzed ECHO and vascular USS studies. BH: Performed and analyzed vascular USS studies particularly TAC and AIX. CH: Conceived the study, participated in statistical analysis and drafting and revising the manuscript JC: Conceived and supervised the oxidative stress component of the study and participated in manuscript revision AM: Carried out the oxidative stress assays, DJ: acquired funding, manuscript revision, SC: Manuscript revision, NI: Acquired funding, manuscript revision, DM: Manuscript revision, TM: Conceived the study, acquired funding, provided overarching supervision, manuscript revision. All authors have read and approved the final manuscript.

## Pre-publication history

The pre-publication history for this paper can be accessed here:

http://www.biomedcentral.com/1471-2369/12/51/prepub
